# Functional, emotional, and physical dimensions of voice fatigue among music teachers at a private arts university in Sichuan, China: a cross-sectional survey

**DOI:** 10.3389/fpubh.2026.1769741

**Published:** 2026-03-17

**Authors:** Zi Zhang, Mei Foong Ang

**Affiliations:** 1 Faculty of Music, Sichuan Film and Television University, Chengdu, Sichuan, China; 2 Department of Music, Faculty of Human Ecology, Universiti Putra Malaysia, Serdang, Selangor, Malaysia

**Keywords:** music teachers, occupational health, private university, voice fatigue, voice fatiguehandicap questionnaire

## Abstract

**Purpose:**

Voice fatigue is a significant occupational hazard for professional voice users, yet it remains within private higher education institutions in China. This study aims to conduct a multidimensional analysis of voice fatigue in music teachers from a single private arts university in Sichuan, China, investigating its functional, emotional, and physical dimensions and their relationship with specific occupational factors in this setting.

**Methods:**

A cross-sectional survey was conducted with 63 teachers at a private arts university in Sichuan, China. Participants completed the validated Voice Fatigue Handicap Questionnaire (MC-VFHQ). Data were analyzed using descriptive statistics, Pearson correlations, and exploratory analysis of variance (ANOVA) to examine the prevalence of voice fatigue and the influence of the pre-specified occupational factor of teaching specialization (course type). No a priori power analysis was conducted; therefore, inferential analyses were interpreted as exploratory and context-specific.

**Results:**

A substantial proportion of teachers (47.6%) reported experiencing voice fatigue “often or always” during work hours. The functional, emotional, and physical dimensions of fatigue were strongly and positively intercorrelated, with the highest correlation found between the functional and physical dimensions (*r* = 0.712, df = 61, *p* = 6.05 × 10^−^11). ANOVA results suggested that physical fatigue differed by course type [*F*_(2, 60)_ = 6.260, *p* = 0.003, η_p^2^ = 0.173]. *Post-hoc* Tukey tests indicated higher physical fatigue among music theory and instrumental instructors than vocal instructors within this sample.

**Conclusion:**

Voice fatigue is a prevalent and multifaceted occupational health issue for the music teachers in the studied institution. The strong interplay between its dimensions and the significant influence of specific teaching duties. These exploratory findings may inform institution-level consideration of targeted voice-health supports in comparable private arts-university settings. Given the modest, non-random, single-site sample and the lack of a priori power analysis, conclusions are limited to this setting and should be treated as hypothesis-generating for broader populations.

## 1 Introduction

### 1.1 Background of the study

The human voice is a fundamental tool for communication, particularly for professionals whose work depends heavily on vocal use. For educators—and music teachers in particular—the voice serves not only as a medium of instruction but also as a key expressive and pedagogical instrument (1). Voice fatigue, defined as a decline in vocal quality and function due to prolonged or intense vocal use, manifests as hoarseness, discomfort, reduced vocal range, and even temporary voice loss (2, 3). The increasing mental stress in modern life, along with unhealthy habits such as irregular schedules and smoking, as well as poor hydration and certain dietary choices known to negatively affect vocal health, have been associated with increased risk of voice problems, especially among professional voice users (4).

Voice disorders have become a frequent complaint in otolaryngology clinics, reflecting a broader concern within vocally demanding professions (5). Teaching has been consistently identified as a high-risk occupation for developing voice disorders due to excessive voice use, poor voice training, high background noise levels, and inadequate working conditions (6). Vocal fatigue, in particular, is among the most commonly reported symptoms, defined as a progressive increase in phonatory effort alongside a decline in vocal performance, often occurring after minutes or hours of voice use (7). Environmental factors such as temperature and classroom acoustics have also been linked to increased voice strain in educators (8).

University music teachers are uniquely susceptible to voice fatigue, given their dual roles as instructors and performers (9). Their teaching duties typically include vocal demonstrations, rehearsals, and performances, all of which require frequent and sustained vocalization (10). Maintaining vocal health is vital not only for professional performance but also for ensuring effective student learning and engagement.

In the context of China's higher education system, private universities—unlike their public counterparts—tend to emphasize teaching over research and impose heavier teaching loads on faculty members (11). National education statistics indicate that private higher education constitutes a substantial component of the higher education landscape in China (12), underscoring the importance of occupational health research in this segment while avoiding assumptions of national representativeness from single-site studies. The unique institutional dynamics of this sector often create high-pressure work environments for academics (13). Furthermore, the rise of managerialism in higher education has been shown to negatively impact teachers' professional wellbeing (13). For instance, at the private arts university examined in this study, the music department alone-though established only in 2017, has grown to serve over 1,800 students. Music educators frequently report teaching loads of 16–20 class sessions per week, in addition to research, rehearsal, and administrative duties. Such demanding conditions can threaten job satisfaction and contribute to professional burnout, which are significant concerns in higher education (14).

Although existing studies have addressed voice disorders among teachers in general, a gap remains in the literature concerning the specific experiences of music educators in China's private institutions. This is a distinct and under-researched group within a large and policy-relevant private higher education segment (12). Their combination of heavy pedagogical loads and the high voice requirements of artistic performance places them in a unique risk profile. In addition, it is important to understand their physical health symptoms because such symptoms are viewed as a major contributor to teacher burnout (15). Thus, the proposed study aims to contribute to new knowledge by offering a quantitative, multidimensional investigation of voice fatigue among music teachers at a private university, exploring the effects of various course types on the functional, emotional, and physical aspects of the phenomenon to inform targeted interventions in this institutional context.

### 1.2 Statement of the problem

Voice plays an essential role in teaching and professional communication. Globally, over one-third of the workforce relies on voice as a critical tool for job performance (16). For teachers, particularly those in music education, the voice is a principal means of instruction. However, extensive and improper use of the voice can lead to fatigue and disorders, reducing teaching quality and job satisfaction (17).

Teachers are significantly more prone to voice-related issues compared to other professionals, with a substantial proportion reporting vocal discomfort such as throat dryness, pain, and hoarseness (18). Common symptoms include hoarseness, vocal fatigue, dry throat, vocal strain, and soreness, all of which are exacerbated by prolonged speaking in noisy or stressful environments (19, 20). Contributing factors include improper vocal techniques, such as inadequate breath control or misuse of vocal mechanisms, as well as psychological stress that may lead to increased vocal tension (21).

The cumulative effects of these factors result in vocal fold fatigue and tissue damage, which compromise vocal performance and wellbeing (4). Voice problems hinder teaching effectiveness, reduce classroom engagement, and can adversely affect interpersonal communication and emotional states. Chronic vocal issues may even compel teachers to take leave or retire prematurely from the profession (22).

Despite the impact of vocal fatigue, the problem remains understudied. In the particular environment of private colleges and universities in China, teachers' class hours are often reported to be significantly higher than those of public colleges and universities. In addition to speaking, singing demonstration and rehearsal after class, music teachers often report experiencing severe vocal exhaustion after a day's teaching, which may affect their perceived work functioning and wellbeing (14, 15). However, these subjective experiences are rarely examined using quantitative instruments in China.

University-level music curricula are generally divided into distinct areas such as vocal performance, instrumental instruction, and music theory. These differences suggest that teachers in each specialization may use their voices in different ways and may therefore experience varying levels of voice fatigue. This distinction underscores the need for targeted studies to inform future voice training programs and occupational health interventions.

Given the growing number of private universities in China and the heavy teaching responsibilities placed on faculty, the vocal health of music teachers deserves more focused scholarly attention. Voice fatigue should be recognized as both an occupational health concern and a quality-of-life indicator. This study addresses this research gap by investigating the prevalence, associated factors, and implications of voice fatigue among music teachers at a private arts university in Sichuan, China, providing a focused examination of this high-demand occupational group without implying representativeness beyond this site.

### 1.3 Research objectives

The primary objectives of this study are three fold:

RO1: To quantify the prevalence and specific manifestations of voice fatigue across its functional, emotional, and physical dimensions among music teachers in a private university in Sichuan, China.
RO2: To examine the relationships among the three dimensions of voice fatigue (functional, emotional, and physical).
RO3: To examine the associations between demographic and occupational variables, particularly course type and years of teaching experience, on the dimensions of voice fatigue as exploratory analyses within this sample.

### 1.4 Research questions

To achieve these objectives, the study addresses the following research questions:

RQ1: What are the prevalence and characteristics of functional, emotional, and physical voice fatigue among the studied private university music teachers?
RQ2: Is there a significant correlation among the functional, emotional, and physical dimensions of voice fatigue?
RQ3: Are occupational factors, specifically course type and years of teaching experience, associated with significant differences in the dimensions of voice fatigue experienced by music teachers in this institution?

### 1.5 Analytical expectations (exploratory)

Based on the existing literature and the theoretical framework of vocal fatigue, the following a priori analytical expectations were specified to guide exploratory analyses:

E1: We expected the functional, emotional, and physical dimensions of voice fatigue to be positively correlated.
E2: We anticipated that occupational demands would vary by specialization, and thus we examined potential differences in physical voice fatigue across course types (vocal performance, instrumental instruction, and music theory).

## 2 Literature review

### 2.1 Introduction

This chapter reviews the literature to establish a theoretical foundation for this study. It defines the multidimensional nature of voice fatigue, contextualizes the participants in this study as a group with heavy voice-use demands, and proposes a theoretical framework specific to music teachers in China's private higher education context.

### 2.2 The multidimensional nature of voice fatigue

Voice fatigue is recognized not merely as a symptom but as a potential precursor to more severe voice disorders, with etiologies ranging from functional to organic (23). At its core, the phenomenon is often linked to vocal hyperfunction—a condition of excessive and/or imbalanced muscle activity in the larynx—which can increase the risk of phonotrauma (24). Prolonged vocal fatigue and its associated compensatory behaviors may lead to chronic conditions such as muscle tension dysphonia, a disorder characterized by strain and discomfort during vocalization (25).

**Figure 1:**
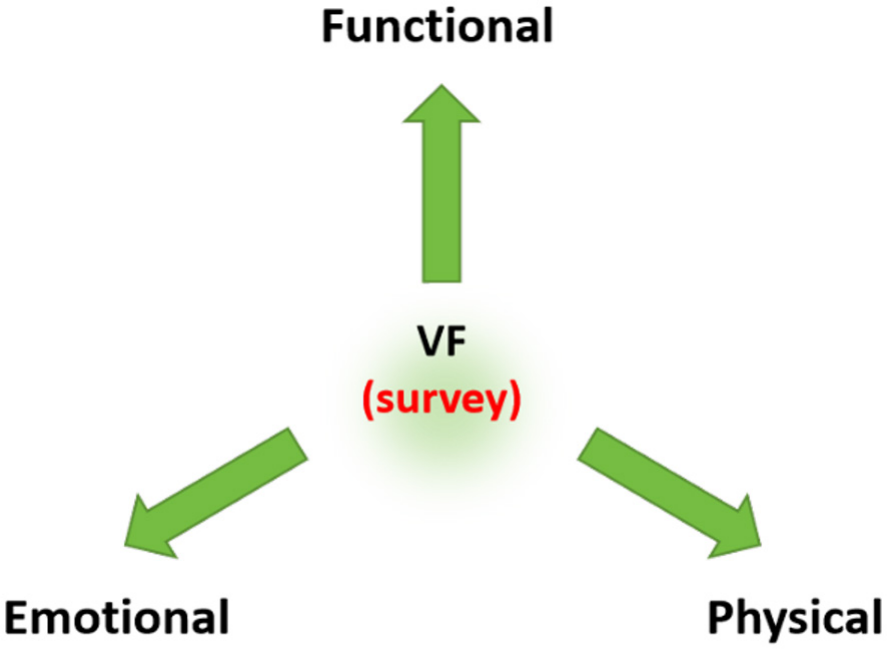
The three-dimensional model of voice fatigue (VF), adapted from Paolillo and Pantaleo (27).

Clinically, vocal fatigue is defined as a decline in vocal function following extended use, encompassing undesirable perceptual, acoustic, and physiological changes (26). However, contemporary understanding has evolved toward a more holistic, multidimensional model. Paolillo and Pantaleo (27) conceptualized voice fatigue as a handicap with three interconnected facets: functional, physical, and emotional (see Figure 1). The functional dimension relates to how fatigue restricts daily vocal tasks; the physical dimension involves somatic symptoms like throat pain and tension; and the emotional dimension captures the psychosocial consequences, such as anxiety and frustration. This multidimensionality is crucial, as recent studies confirm a significant relationship between vocal fatigue and a reduced overall quality of life among professional voice users (28).

### 2.3 Educators as a high-risk population

Teaching has long been identified as a high-risk profession for voice-related problems. Seminal studies established that teachers experience voice disorders at significantly higher rates than the general population, affecting their work performance, attendance, and career longevity (20, 29). This elevated risk remains a global concern, with recent international studies continuing to report high prevalence rates; for instance, a 2024 study in Saudi Arabia confirmed that a majority of teachers experienced voice disorders and identified key risk factors including high weekly class loads (30).

The primary driver for this risk is the exceptionally high occupational vocal load. Recent research utilizing objective voice dosimetry has begun to quantify the extensive daily phonation times and intensity levels required of teachers, confirming that their vocal use often exceeds that of other professions (31). A 2024 study specifically on university professors further corroborated these findings of high vocal load within the higher education context (32). This sustained vocal demand is often compounded by poor classroom acoustics and high background noise, forcing teachers to further increase their vocal effort. In the specific context of China, these occupational demands are situated within a high-pressure work environment that has been shown to contribute to significant job stress and professional burnout among educators in higher institutions (33, 34). Consequently, the wellbeing of teachers is an area of growing concern, where physical symptoms like voice fatigue can be both a cause and a consequence of broader occupational stress (35).

### 2.4 A theoretical framework for voice fatigue in music teachers

**Figure 2:**
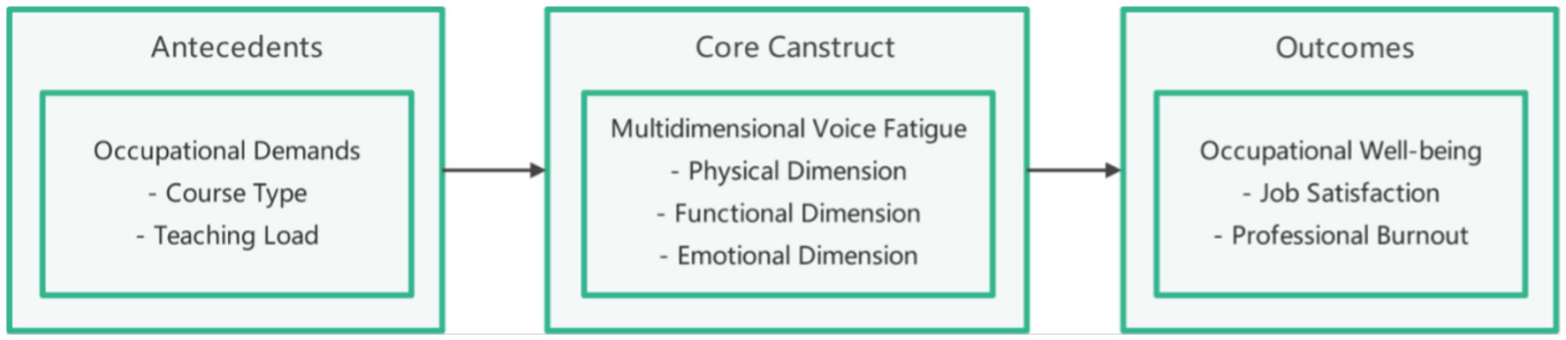
Theoretical Framework (hypothesized pathway; “occupational wellbeing” outcomes not measured in this survey).

While the literature establishes general risks for educators, music teachers require a specific conceptual framework due to their unique professional demands. As shown in Figure 2 (Theoretical Framework), this study proposes a theoretical framework linking specific occupational demands to the multidimensional experience of voice fatigue and hypothesized downstream consequences. Importantly, the present survey did not measure “occupational wellbeing” outcomes; this pathway is presented as a theoretical guide for future research rather than an empirically tested model in this dataset.

The framework begins with Specific Occupational Demands as the primary antecedent. For music teachers, this includes the high baseline vocal load of all educators, plus discipline-specific tasks like frequent singing demonstrations and rehearsals which demand precise and sustained vocal control (32). These heightened demands can trigger maladaptive vocal behaviors consistent with models of Vocal Hyperfunction (24), leading to the primary outcome: Multidimensional Voice Fatigue, experienced across the physical, functional, and emotional dimensions (27). Finally, this chronic state of fatigue is proposed to negatively impact teachers' Occupational Wellbeing, a link supported by broader research connecting persistent physical health symptoms to professional burnout (15, 33).

### 2.5 Summary and research gap

In summary, the literature establishes voice fatigue as a multidimensional problem rooted in vocal hyperfunction, particularly for high-risk groups like educators. However, a significant gap remains. While the link between teaching and voice problems is well-documented, few studies have quantitatively investigated how specific pedagogical tasks differentially impact the multidimensional experience of voice fatigue, especially within the unique context of music education. Recent reviews highlight the need for research that can inform targeted prevention strategies for specific occupational groups (36). Furthermore, research on the occupational vocal health of educators in China's large private higher education context is scarce. This study aims to address this gap by providing a quantitative, multidimensional analysis of voice fatigue among music teachers at a private arts university in Sichuan, China, thereby laying the groundwork for evidence-based occupational health interventions in similar settings.

## 3 Methodology

### 3.1 Research design

This study employed a quantitative, cross-sectional survey design, using a structured online questionnaire distributed via the Questionnaire Star (WJX.cn) platform to investigate the multidimensional nature of voice fatigue among music teachers at the studied private arts university in Sichuan, China.

### 3.2 Participants and sampling

This study used a single-institution design. The study site (Sichuan Film and Television University, a private arts university in Sichuan, China) was selected for feasibility and contextual relevance. Within the institution, we adopted an attempted census approach by inviting all music faculty members in the Music Department (*N* = 73) to participate. While the final sample of 63 participants represents a high response rate, no a priori power analysis was conducted. Given the modest sample size and single-site design, the study is best viewed as exploratory. The findings—particularly subgroup comparisons—should be interpreted with appropriate caution, as limited statistical power may constrain the detection of small to moderate effects.

### 3.3 Instrument

The Voice Fatigue Handicap Questionnaire (VFHQ), a validated 30-item tool, was used to assess the functional, emotional, and physical dimensions of vocal fatigue (27). To ensure its linguistic and cultural validity for the Chinese context, We used the validated Mandarin Chinese version of the VFHQ (MC-VFHQ) (37) for identifying individuals potentially experiencing voice fatigue. It demonstrates sound reliability, validity, and sensitivity when administered to Mandarin-speaking populations.

In the current study, the instrument demonstrated high internal consistency, with a Cronbach's α was 0.939 for the total scale. Reliability coefficients for the three subscales acceptable to excellent internal consistency (α = 0.698–0.929).

### 3.4 Data analysis

Data were analyzed using IBM SPSS Statistics 27.0. Descriptive statistics (frequencies, means, *SD*) were used to summarize demographics and VFHQ scores. Pearson's correlation coefficient (*r*) was used to explore associations among the three VFHQ dimensions (functional, emotional, and physical). Pearson correlations were restricted to continuous VFHQ subscale scores and were not applied to nominal variables. One-way ANOVA was used to explore group differences by the nominal factor course type (vocal performance, instrumental instruction, music theory). Course type was coded for data management (1 = vocal, 2 = instrumental, 3 = theory) but was analyzed as a categorical factor, not as a continuous variable. Prior to conducting the ANOVA, the assumption of homogeneity of variances was tested using Levene's test. For significant ANOVA results, Tukey's HSD *post-hoc* tests were performed. Effect sizes (Pearson's r and partial eta-squared, η*p*^2^) were calculated for all key analyses to determine the magnitude of observed differences. Given the absence of an a priori power analysis, inferential tests were interpreted as exploratory and aimed at identifying potentially meaningful associations or group differences rather than confirming hypotheses. Statistical significance was set at *p* < 0.05. Given the exploratory nature of the study and the use of multiple statistical tests, results were interpreted cautiously without formal adjustment for multiplicity.

## 4 Results and data analysis

### 4.1 Descriptive statistics of voice fatigue handicap questionnaire (VFHQ)

A total of 73 questionnaires were distributed to faculty members in the Music Department of the studied private university. Sixty-six responses were collected, among which four were excluded due to issues identified during data cleaning. The criteria for identifying invalid responses included highly repetitive answer patterns, regular or mechanical response sequences, and completion time under 66 s, the completion-time threshold was set based on the pilot median completion time. After screening, 63 valid questionnaires were retained for analysis (Table 1). The final instrument demonstrated excellent internal consistency (Cronbach's α = 0.939).

**Table 1:** Demographic characteristics of participants.

**Factor**	**Category**	**Frequency (*n*)**	**Percentage (%)**
Gender	Female	47	74.6%
	Male	16	25.4%
Age	≦30	33	52.4%
	31–40	24	38.1%
	>40	6	9.5%
Course category	Vocal	28	44.4%
	Instrumental	18	28.6%
	Music theory	17	27.0%
Years of service	≦5	35	55.6%
	6–10	18	28.5%
	11–20	7	11.1%
	>20	3	4.8%

Among the 63 valid participants, the majority were female (74.6%). The average age was 32.71 years (*SD* = 7.56), with a majority aged under 31 (52.4%), followed by those aged 31–40 (38.1%). Vocal teachers represented the largest group (44.4%), followed by instrumental (28.6%) and music theory instructors (27.0%). The majority of respondents (55.6%) had fewer than six years of teaching experience (*M* =7.27, *SD* = 6.12), reflecting a relatively young teaching cohort.

The descriptive statistics for the three dimensions of voice fatigue are presented below. The Functional dimension had a mean score of 2.54 (*SD* = 0.52, Range 1.30–3.50). The Emotional dimension showed a mean of 2.13 (*SD* = 0.65, Range 1.00–3.80), while the Physical dimension had a mean of 2.20 (*SD* = 0.82, Range 1.00–5.00). These scores indicate that, on average, the participants experienced low to moderate levels of voice fatigue handicap across these domains.

#### 4.1.1 (1) Occurrence and timing of voice fatigue

Supplementary Table S1 illustrates the distribution of self-reported voice fatigue throughout the day and across settings. The results indicate that voice fatigue predominantly occurs during work hours and tends to worsen by the end of the day. Nearly half of the participants (47.6%) reported experiencing voice fatigue often or always while working, whereas only 4.8% reported frequent symptoms during non-working hours. This indicates that voice fatigue is predominantly associated with occupational vocal load.

#### 4.1.2 (2) Behavioral responses to voice fatigue

Supplementary Table S1 indicates that most participants did not engage in aggressive coping strategies. Few teachers reported straining their voice (9.5%) or taking medication (3.2%). However, many changed their vocal habits (17.4%) or spoke less socially (12.7%) to cope with fatigue. These findings indicate that most participants adopted minimal coping strategies despite experiencing symptoms of fatigue.

#### 4.1.3 (3) Emotional reactions and voice anxiety

Supplementary Table S1 shows that while most participants did not report persistent emotional distress related to voice fatigue, approximately 14.3% reported moderate to high concern regarding voice deterioration during the day and its impact on work performance. Overall, voice fatigue-related anxiety—when present—was primarily work-oriented rather than social-life oriented in this sample.

#### 4.1.4 (4) Impact on daily life

Voice fatigue had the most pronounced impact on work-related tasks, with 14.3% of participants indicating they often or always felt impaired at work due to voice fatigue. In contrast, perceived impact on social (4.8%) and domestic (6.3%) contexts was relatively low (Supplementary Table S1).

#### 4.1.5 (5) Impact of voice-related anxiety

A small proportion of participants expressed emotional or social distress due to voice-related anxiety, although the majority did not perceive a significant impact on their wellbeing or interpersonal interactions (Supplementary Table S1).

#### 4.1.6 (6) Voice symptomatology

The most commonly reported symptoms were hoarseness, dull vocal quality, and vocal strain, all of which may indicate early manifestations of vocal fatigue or overuse (Supplementary Table S1).

#### 4.1.7 (7) Physiological manifestations

Supplementary Table S1 indicates that physical symptoms, such as neck and shoulder tension (27.0%), were more frequently endorsed than vocal instability (9.7%) or throat tightness (14.3%), suggesting that musculoskeletal strain may be relevant to perceived voice fatigue in this sample.

### 4.2 Correlation analysis

Subscale mean scores were computed for the functional, emotional, and physical domains of the VFHQ, and Pearson correlations were then calculated among these continuous subscale scores. The results revealed statistically significant positive correlations among them (Table 2). Specifically, the functional dimension was significantly and positively correlated with the emotional dimension (*r* = 0.685, *df* = 61, *p* = 5.94 × 10^−10^). Furthermore, the functional dimension was positively correlated with the physical dimension (*r* = 0.712, *df* = 61, *p* = 6.05 × 10^−11^). Likewise, the emotional and physical dimensions were also significantly positively correlated (*r* = 0.664, *df* = 61, *p* = 2.99 × 10^−9^). These findings support a strong interrelationship among the three dimensions of voice fatigue in this sample.

**Table 2:** Pearson correlation among functional, emotional, and physical dimensions with df and exact *p*-values.

**Dimension pair**	**r**	**df**	***p* value**
Functional–emotional	0.685	61	5.94 × 10^−10^
Functional–physical	0.712	61	6.05 × 10^−11^
Emotional–physical	0.664	61	2.99 × 10^−9^

### 4.3 ANOVA analysis

A one-way ANOVA was conducted to explore whether teaching specialization (course type) was associated with differences in VFHQ subscale scores. Given the modest subgroup sizes and the lack of a priori power analysis, these comparisons were interpreted as exploratory.

Levene's test indicated that the assumption of homogeneity of variances was met (*p* > 0.05). The analysis suggested a statistically significant difference in the physical dimension of voice fatigue among course types [*F*_(2, 60)_ = 6.260, *p* = 0.003, η_p^2^ = 0.173] (Table 3). Tukey's HSD *post-hoc* tests indicated that music theory instructors reported higher physical fatigue than vocal instructors (*p* = 0.002), and instrumental instructors also reported higher physical fatigue than vocal instructors (*p* = 0.009). The difference between music theory and instrumental instructors was not statistically significant (*p* > 0.05). Because potential explanatory variables such as class size, objective vocal load, classroom acoustics, smoking, and hydration were not measured in this survey, mechanisms underlying these subgroup differences cannot be determined from the current dataset.

**Table 3:** Exploratory ANOVA result for physical dimension by course type (key model).

**Factor**	**Outcome**	**df_between**	**df_within**	***F***	***P***	**η_p^2^**
Gender	Functional	–	–	–	>0.05	–
	Emotional	–	–	–	>0.05	–
	Physical	–	–	–	>0.05	–
Age	Functional	–	–	–	>0.05	–
	Emotional	–	–	–	>0.05	–
	Physical	–	–	–	>0.05	–
Years of teaching	Functional	–	–	–	>0.05	–
	Emotional	–	–	–	>0.05	–
	Physical	–	–	–	>0.05	–
Course type	Physical	2	60	6.260	0.003	0.173

**Figure 3:**
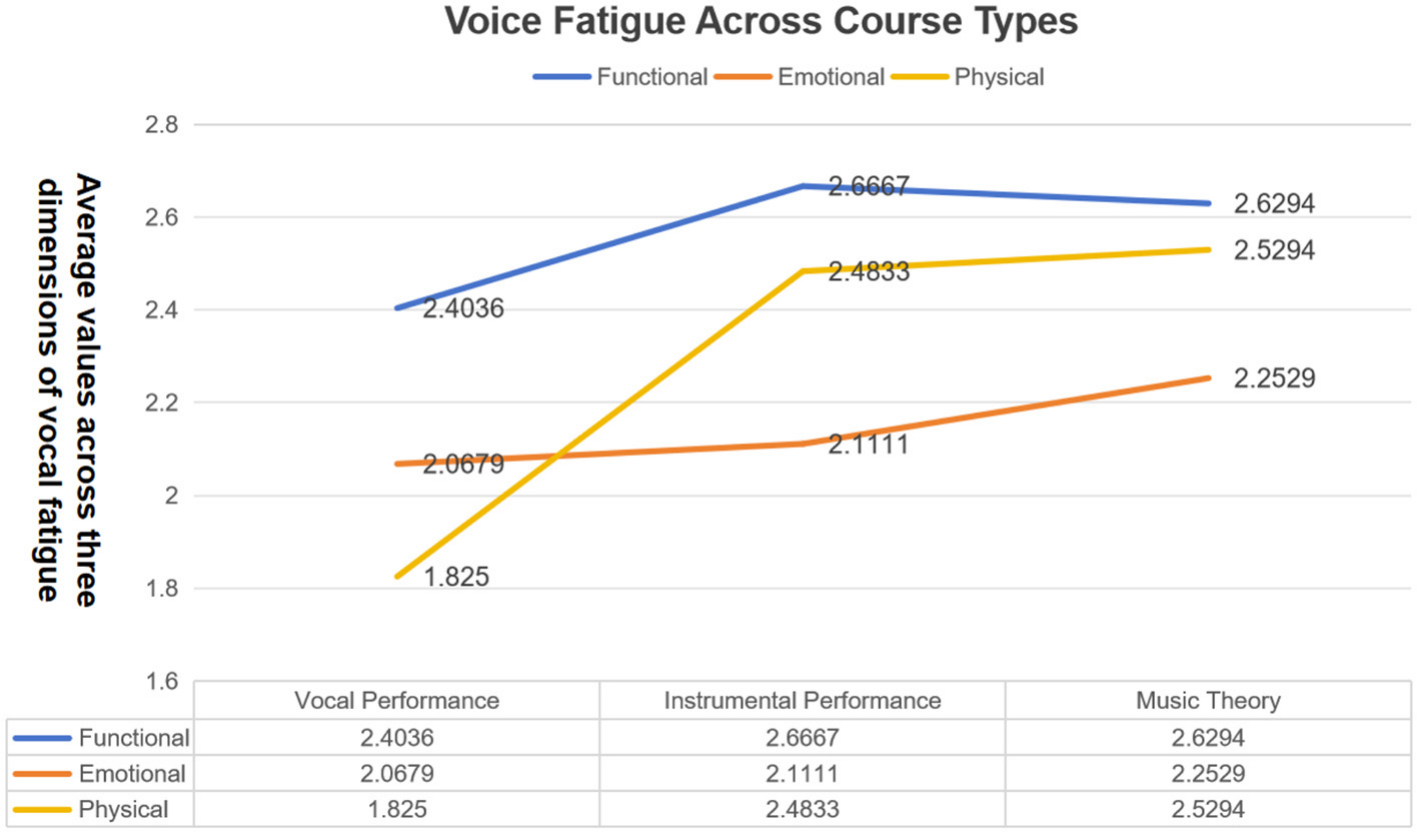
Comparison of mean values across three dimensions of vocal fatigue in different course types.

As illustrated in Figure 3, mean values for the three dimensions are compared across course types.

### 4.4 Summary of findings

This study analyzed the prevalence and multidimensional characteristics of vocal fatigue among music teachers at the studied private arts university in Sichuan, China. Findings indicate that a substantial proportion of participants reported frequent work-related voice fatigue, and the functional, emotional, and physical dimensions of fatigue were strongly positively correlated.

Teaching specialization (course type) was associated with differences in physical voice fatigue, with music theory and instrumental teachers reporting higher physical fatigue than vocal teachers in this sample. This pattern suggests that course-related occupational demands may be relevant to physical manifestations of voice fatigue; However, such interpretations remain hypotheses because related workload and environmental variables were not measured. However, explanations involving class size, posture, or acoustics remain hypotheses because these variables were not measured. Behaviorally, many teachers reported may indicate coping strategies which should be examined in future research, suggesting potential gaps in resources or awareness related to vocal health.

Overall, the study suggests that voice fatigue is a prevalent and multifaceted occupational challenge among music teachers in the studied institution. These results provide a basis for future multi-site research and for developing context-appropriate vocal health support and prevention strategies.

## 5 Discussion

### 5.1 Interpretation and implications

This study's findings suggest that voice fatigue is a prevalent occupational health issue among music teachers at the studied private university, aligning with both classic literature identifying educators as a high-risk group (20, 29) and recent international surveys (18). The primary contribution of this research is its multidimensional analysis. The strong positive correlation found among the functional, emotional, and physical dimensions provides empirical support for a holistic model, suggesting that interventions targeting only physical symptoms without addressing functional and emotional distress may be insufficient.

Crucially, the results revealed that the type of course taught was associated with physical fatigue scores in this sample. This finding should be interpreted as exploratory and hypothesis-generating, given the modest subgroup sizes and the absence of a priori power analysis. While it is plausible that differences in teaching format or vocal demands across specializations could contribute to this pattern, the current survey did not measure candidate explanatory factors (e.g., class size, vocal dose, classroom noise, posture), and therefore such interpretations should be treated as hypotheses rather than evidence-based mechanisms.

Emotionally, a minority of teachers reported voice-related anxiety, primarily concerning work performance. Behaviorally, many teachers reported limited coping strategies (e.g., low rates of medication use and relatively modest behavioral adjustments), suggesting a potential need for institutional education and structured support mechanisms.

Despite the physical and psychological burden, there appears to be limited engagement with preventive or remedial strategies, underscoring the need for systematic vocal health education and institutional intervention programs. Because occupational wellbeing outcomes were not measured, the present study cannot quantify downstream effects on wellbeing; this relationship is presented in Figure 2 as a theoretical pathway for future research (38).

### 5.2 Practical implications

These findings have practical implications for administrators in the studied institution and similar private higher education settings. The results suggest that workload management and voice health support might consider course-type demands alongside conventional scheduling metrics. For example, scheduling policies could avoid assigning vocally intensive classes back-to-back. Institutions may also consider offering vocal hygiene education and facilitating access to screening or consultation for teachers who report frequent voice fatigue.

### 5.3 Limitations of the study

The study's findings should be interpreted in light of its limitations. First, the single-institution, purposive sample limits generalizability; results should not be interpreted as representative of all music teachers in China or of the entire private higher education sector. Second, the modest sample size (*N* = 63) and small subgroup cell sizes reduce statistical power, particularly for subgroup comparisons, increasing the risk of Type II error and potentially yielding unstable effect-size estimates. Third, no a priori power analysis was conducted, multiple statistical tests were conducted and were interpreted in an exploratory manner without formal adjustment for multiplicity.. Fourth, all outcomes relied on self-report, which may be influenced by recall bias and social desirability bias. Fifth, potential confounders and risk modifiers—including smoking, hydration, medication use, reflux symptoms, and other health factors—were not measured and could not be controlled. Finally, no objective indicators of vocal load (e.g., voice dosimetry, acoustic measures, or classroom noise) or clinical laryngeal assessments were collected; therefore, associations cannot be linked to quantified vocal exposure or physiological mechanisms. In addition, the cross-sectional design precludes causal inference.

### 5.4 Directions for future research

Future research should build upon this study by employing multi-site designs, larger samples, and objective measures such as voice dosimetry or acoustic monitoring to quantify vocal load across teaching specializations. Collecting key confounders (e.g., smoking, hydration) and classroom/environmental measures would enable more robust covariate adjustment and facilitate stronger causal inference when combined with longitudinal or quasi-experimental approaches. Additionally, qualitative studies may help explore the lived experience behind emotional reactions to voice fatigue and inform tailored preventive programs.

## 6 Conclusion

This study suggests voice fatigue as a common occupational health problem among the music educators at the studied private arts university in Sichuan, China. The results show significant positive associations among the functional, emotional, and physical aspects of voice fatigue, and teaching specialization was associated with differences in physical fatigue. Because the design is cross-sectional and the sample is non-random and single-site, conclusions are limited to this setting and should be treated as exploratory. The findings may inform institution-level of targeted, context-appropriate voice health support and motivate future multi-site research incorporating objective vocal load measures and broader occupational outcomes.

## Data Availability

The raw data supporting the conclusions of this article will be made available by the authors, without undue reservation.
