# Supplementary material for: Functional, emotional, and physical dimensions of voice fatigue among music teachers at a private arts university in Sichuan, China: a cross-sectional survey
Source: Front Public Health. 2026 Mar 17;14:1769741. doi: 10.3389/fpubh.2026.1769741 (PMC13036135; doi:10.3389/fpubh.2026.1769741)
Supplement: Supplementary file 1 [file Table_1.docx]

**Functional, emotional, and physical dimensions of voice fatigue among music teachers at a private arts university in Sichuan, China: a cross-sectional survey**

Zi Zhang^1,2^，Mei Foong Ang^2*^

^1^ Sichuan Film and Television University, Chengdu, China.

^2^ Universiti Putra Malaysia, Serdang Selangor Darul Ehsan, Malaysia

^*^ Corresponding author: Mei Foong Ang, E-mail Address: meifoong@upm.edu.my

**Supplementary Table S1. Item-level descriptive statistics (often/always frequency; mean; SD) for all VFHQ items (n = 63).**

| **Theme** | **Item** | **Often/always (n)** | **%** | **Mean** | **SD** |
| --- | --- | --- | --- | --- | --- |
| Occurrence/Timing | VF appears at the beginning of the day | 10 | 15.9 | 2.29 | 1.09 |
| Occurrence/Timing | VF appears as the day progresses | 16 | 25.4 | 2.86 | 0.96 |
| Occurrence/Timing | VF appears at the end of the day | 20 | 31.7 | 3.00 | 1.03 |
| Occurrence/Timing | VF appears mostly at work | 30 | 47.6 | 3.48 | 1.03 |
| Occurrence/Timing | VF appears outside work | 3 | 4.8 | 1.90 | 0.85 |
| Coping/Behavior | Strain to produce voice | 6 | 9.5 | 1.98 | 1.08 |
| Coping/Behavior | Throat clearing | 12 | 19.1 | 2.63 | 1.02 |
| Coping/Behavior | Change vocal/behavioral habits | 11 | 17.4 | 2.78 | 1.02 |
| Coping/Behavior | Speak less socially | 8 | 12.7 | 2.51 | 0.87 |
| Coping/Behavior | Use medication | 2 | 3.2 | 1.97 | 0.87 |
| Emotional reaction/anxiety | Speak less due to fear of damage | 6 | 9.5 | 1.56 | 0.71 |
| Emotional reaction/anxiety | Feel not at full vocal capacity | 7 | 11.1 | 2.49 | 0.859 |
| Emotional reaction/anxiety | Fear worsening of voice throughout the day | 8 | 12.7 | 2.41 | 0.90 |
| Emotional reaction/anxiety | Anxiety about impact of voice on job performance | 9 | 14.3 | 2.54 | 0.93 |
| Functional limitation | Feel handicapped at work | 9 | 14.3 | 2.59 | 0.87 |
| Functional limitation | Feel handicapped in social or personal life | 3 | 4.8 | 1.94 | 0.84 |
| Functional limitation | Feel handicapped at home | 4 | 6.3 | 1.81 | 0.91 |
| Psychological effects | Voice anxiety affects emotions | 4 | 6.4 | 2.08 | 0.92 |
| Psychological effects | Voice anxiety affects lifestyle | 4 | 6.4 | 1.89 | 0.90 |
| Psychological effects | Others do not understand the vocal burden | 6 | 9.5 | 2.00 | 0.95 |
| Speaking voice symptoms | Voice breaks | 5 | 8.0 | 1.87 | 0.95 |
| Speaking voice symptoms | Difficulty controlling voice | 8 | 12.7 | 2.16 | 1.11 |
| Speaking voice symptoms | Voice becomes weak | 3 | 4.8 | 2.14 | 0.89 |
| Speaking voice symptoms | Voice becomes breathy | 7 | 11.1 | 2.02 | 1.05 |
| Speaking voice symptoms | Voice becomes hoarse | 8 | 12.7 | 2.27 | 1.08 |
| Speaking voice symptoms | Voice becomes strained | 3 | 4.8 | 2.08 | 0.97 |
| Speaking voice symptoms | Voice becomes duller/less vibrant | 7 | 11.1 | 2.19 | 1.05 |
| Physical symptoms | Tightness in the throat | 9 | 14.3 | 2.24 | 1.10 |
| Physical symptoms | Discomfort/tension/pain in neck and shoulders | 17 | 27.0 | 2.71 | 1.27 |
| Physical symptoms | Vocal instability throughout the day | 6 | 9.5 | 2.35 | 0.98 |
